# Health economic evaluation of Human Papillomavirus vaccines in women from Venezuela by a lifetime Markov cohort model

**DOI:** 10.1186/s12889-017-4064-7

**Published:** 2017-02-02

**Authors:** Ariel Esteban Bardach, Osvaldo Ulises Garay, María Calderón, Andrés Pichón-Riviére, Federico Augustovski, Sebastián García Martí, Paula Cortiñas, Marino Gonzalez, Laura T. Naranjo, Jorge Alberto Gomez, Joaquín Enzo Caporale

**Affiliations:** 1 IECS Instituto de Efectividad Clínica y Sanitaria, Dr. Emilio Ravignani 2024 (C1014CPV), 1014 Buenos Aires, Argentina; 2 Salud Chacao, Final Av. Ávila, Edif. Salud Chacao. Urb. Bello Campo. Chacao, 1060 Caracas, D.C Venezuela; 3 Unit of Public Policy, Simon Bolivar University, Edificio Física y Electrónica I, Planta Baja. Valle de Sartenejas, Estado Miranda Caracas, Venezuela; 4 GlaxoSmithKline Biologicals, Clayton, Ciudad del Saber Edificio 230, Panama City, Panama; 5 GSK Vaccines Latin America, Carlos Casares 3690, B1644 BCD Victoria, Buenos Aires Argentina

**Keywords:** HPV vaccines, Cervical cancer, Genital warts, Health economic evaluation, Markov cohort model, Venezuela

## Abstract

**Background:**

Cervical cancer (CC) and genital warts (GW) are a significant public health issue in Venezuela. Our objective was to assess the cost-effectiveness of the two available vaccines, bivalent and quadrivalent, against Human Papillomavirus (HPV) in Venezuelan girls in order to inform decision-makers.

**Methods:**

A previously published Markov cohort model, informed by the best available evidence, was adapted to the Venezuelan context to evaluate the effects of vaccination on health and healthcare costs from the perspective of the healthcare payer in an 11-year-old girls cohort of 264,489. Costs and quality-adjusted life years (QALYs) were discounted at 5%. Eight scenarios were analyzed to depict the cost-effectiveness under alternative vaccine prices, exchange rates and dosing schemes. Deterministic and probabilistic sensitivity analyses were performed.

**Results:**

Compared to screening only, the bivalent and quadrivalent vaccines were cost-saving in all scenarios, avoiding 2,310 and 2,143 deaths, 4,781 and 4,431 CCs up to 18,459 GW for the quadrivalent vaccine and gaining 4,486 and 4,395 discounted QALYs respectively. For both vaccines, the main determinants of variations in the incremental costs-effectiveness ratio after running deterministic and probabilistic sensitivity analyses were transition probabilities, vaccine and cancer-treatment costs and HPV 16 and 18 distribution in CC cases. When comparing vaccines, none of them was consistently more cost-effective than the other. In sensitivity analyses, for these comparisons, the main determinants were GW incidence, the level of cross-protection and, for some scenarios, vaccines costs.

**Conclusions:**

Immunization with the bivalent or quadrivalent HPV vaccines showed to be cost-saving or cost-effective in Venezuela, falling below the threshold of one Gross Domestic Product (GDP) per capita (104,404 VEF) per QALY gained. Deterministic and probabilistic sensitivity analyses confirmed the robustness of these results.

**Electronic supplementary material:**

The online version of this article (doi:10.1186/s12889-017-4064-7) contains supplementary material, which is available to authorized users.

## Background

Cervical cancer (CC) is the fourth most common cancer in women worldwide with an estimated toll of 528,000 new cases and 266,000 deaths in 2012 [1]. In Latin America and the Caribbean region (LAC) the age-standardized incidence rates ranged from 14.6 to 44.0 per 100,000 women per year, values probably underestimated due to insufficient coverage and frequency of screening, inadequate collection and analysis of cytological samplings as well as incomplete follow-up of cases [2, 3]. The Bolivarian Republic of Venezuela is a South-American country with a population of 27.2 million people where 1 in 3 women is aged 15 years and older and are at risk of developing CC [4]. In this country, CC is the first most common female cancer in women aged 15 to 44 years, accounting for 1,973 new cases diagnosed in 2011 and 1,789 new CC-related deaths annually for all ages [5, 6].

The Human Papillomavirus (HPV) infection occurs commonly in sexually active women; it has been identified as the necessary cause of CC and has been associated with other types of cancer [7–9]. High-risk types of HPV (HPV 16, 18, 45, 31, 33, 39, 52, 58 and 35) have been recognized as a necessary etiological agent for the development of CC and premalignant cervical lesions, being detected in up to 99.7% of such cases [10–13]. Low-risk HPV types (6, 11, 34, 40, 42, 43 and 44) have been associated with genital warts (GW) and low-grade cervical lesions. Among Venezuelan women, HPV types 16 and 18 were identified as the most common high-risk HPV types with an overall prevalence of 80% in patients with CC [14, 15]. It is established that CC comprehensive prevention approaches, including well-organized cervical screening programs using Papanicolaou smear tests (Pap), can reduce CC incidence and mortality [16]. Additionally, the introduction of HPV vaccination is expected to also reduce the burden of CC [17]. Currently, there are two available vaccines: a bivalent vaccine targeting high-risk HPV 16 and 18 (*Cervarix*™, GSK) and quadrivalent vaccine targeting, in addition to the above, also low-risk HPV 6 and 11 types (*Gardasil*, Merck) [18]. Both vaccines have proven efficacy in the prevention of lesions associated with the HPV types they target [19, 20].. Currently in Venezuela, there is a nationwide CC screening program including Paps for women of 25 to 65 years old. However, the HPV vaccine has not yet been incorporated into the national vaccination program and consequently, there is no vaccination coverage [5, 21].

Cost-effectiveness analysis is a useful tool to assist decision makers in assessing the value of specific interventions and inform resource-allocation decisions. Several models based on economic evaluations have been conducted to assess the cost-effectiveness of interventions that reduce HPV-associated premalignant and malignant lesions in Latin America, including vaccination [22, 23]. The results of the analyses vary widely from country to country due to differences in epidemiological factors such as the incidence of certain HPV-related diseases, local treatment patterns and related costs [22]. Therefore, a country-specific assessment needs to be conducted in order to suitably inform local decision-makers.

The aim of the present study was to perform an economic evaluation based on a cohort model adapted to estimate the lifetime cost and clinical outcomes of vaccination with the bivalent and quadrivalent vaccination schemes, comparing them with the current policy in Venezuela of no vaccination.

## Methods

### Model structure

**Fig. 1:**
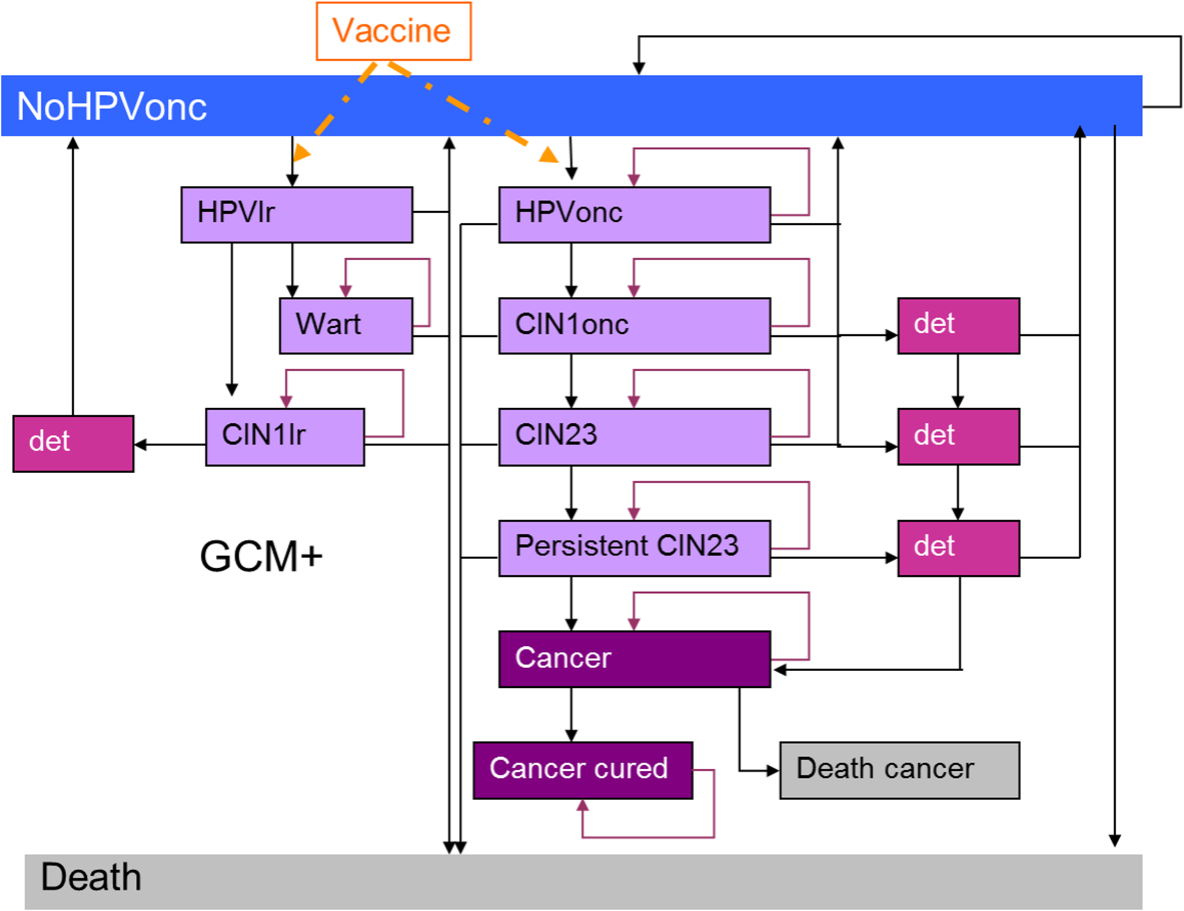
Model structure. Abbreviations: GCM+: Gardasil-Cervarix Model; CIN1onc, cervical intraepithelial neoplasia 1 oncogenic; CIN1lr, low-risk cervical intraepithelial neoplasia 1; det, CIN health state detected through screening: same pathways as CIN nondetected CIN health state but with different probabilities; HPV, human papillomavirus; HPVlr, low-risk HPV infection; HPVonc, oncogenic HPV infection; NoHPVonc, no oncogenic HPV infection, CIN2/3, cervical intraepithelial neoplasia 2 or 3

A previously published lifetime Markov cohort model with a 1-year cycle was adapted to reflect the Venezuelan setting. The model simulated the natural history of oncogenic HPV in CC, the effect of screening and the impact of vaccination over the lifetime of the cohort. [24–26] The current model has been extended to include infection with low-risk HPV types (HPV 6 and HPV 11) that might progress to cervical intraepithelial neoplasia grade 1 (CIN1) and/or to GW (Fig. 1) [18, 27]. The perspective was stated as of the healthcare payer including direct medical costs only. The analysis allowed the estimation of costs and effects of each of the three intervention strategies: current screening program, current screening program in addition to vaccination with the bivalent vaccine, and current screening program in addition to vaccination with the quadrivalent vaccine. For this analysis, a lifetime horizon was adopted. In order to reflect the range of possible results taking into account different vaccine costs, alternative exchange rates for Venezuela, and 2-dose schedule vs. 3-dose schedule, eight scenarios were considered for the base-case analyses, as detailed in the following sections.

### Input parameters

An extensive review of the literature was done in MEDLINE and LILACS. Local information related to epidemiology, use of resources and costs associated to HPV-lesions treatment was collected. Priority to local sources of information was given. Selected parameters were used to populate the model and were reviewed and validated by experts. Input parameters included are summarized in Table 1.

**Table 1:** Input data values: base-case values, associated ranges, probabilities distributions assumed and sources

Inputs	Value (range)		Source
Population data			
11-years old women cohort	264,489	-	[4]
Transition probabilities			
HPV onc regression	0.50 (0.23; 0.77)	Uniform (0.23; 0.77)	[18, 64]
HPV onc to CIN1 progression	0.05 (0.03; 0.07)	Normal (0.03; 0.07)	[18, 64]
CIN1 onc regression	0.36 (0.22; 0.78)	Normal (0.22; 0.78)	[18, 64]
CIN1 onc to CIN2/3 progression	0.14 (0.08; 0.16)	Normal (0.08; 0.16)	[18, 64]
CIN2/3 regression to no HPV	0.25 (0.23; 0.32)	Normal (0.23; 0.32)	[18, 64]
CIN2/3 progression to cancer	0.14 (0.10; 0.15)	Normal (0.10; 0.15)	[18, 64]
HPV low-risk CIN1	0.04 (0.03; 0.05)	Normal (0.03; 0.05)	[18, 64]
Genital wart resistance	0.35 (0.28; 0.42)	Uniform (0.28; 0.42)	[65]
Proportion CIN1 onc detected and treated	0.30 (0.24; 0.36)	Uniform (0.24; 0.36)	Exp. Op
CIN1 treatment success	0.94 (0.75; 1.00)	Uniform (0.75; 1.00)	[35]
Proportion CIN2/3 detected and treated	0.90 (0.72; 1.00)	Uniform (0.72; 1.00)	Exp. Op
CIN2/3 treatment success	0.88 (0.70; 1.00)	Uniform (0.70; 1.00)	[35]
Utility weights			
No HPV	1.000	-	[18]
HPV	1.000	-	[18]
Genital Wart	0.02 (0.02; 0.02)	Uniform (0.02; 0.02)	[18]
CIN1	1.000	-	[18]
CIN1 detected	0.01 (0.01; 0.02)	Uniform (0.01; 0.02)	[18]
CIN2/3	1.000	-	[18]
CIN2/3 detected	0.01 (0.01; 0.01)	Uniform (0.01; 0.01)	[18]
Cancer	0.27 (0.22; 0.33)	Uniform (0.22; 0.33)	[18]
Cancer cured	0.06 (0.05; 0.07)	Uniform (0.05; 0.07)	[18]
Death	0.000	-	[18]
Screening Characteristics			
Regular screening coverage	35%	-	[21]
Interval between regular screening	3 years (& scenario of 5y)	-	[37]
Irregular screening coverage	25%	-	Exp. Op
Population without screening	40%	-	Exp. Op
Age of initiation of screening	25 years	-	[21]
Sensitivity to detect CIN1	0.58 (0.38; 0.56)	Normal (0.38; 0.56)	[22]
Sensitivity to detect CIN2 and CIN3	0.61 (0.69; 0.87)	Normal (0.69; 0.87)	[22]
Estimated positive Pap smear	0.05 (0.05; 0.06)	Uniform (0.04; 0.06)	Exp. Op
Parameters to estimate vaccine effectiveness			
Prevalence of HPV types 16 and 18 in cervical cancer	0.80 (0.64; 0.96)	Uniform (0.64; 0.96)	[15]
Prevalence of other oncogenic HPV in cervical cancer	0.16 (0.13; 0.19)	Uniform (0.13; 0.19)	[15]
Prevalence of HPV types 16 and 18 in CIN2/3	0.55 (0.44; 0.66)	Uniform (0.44; 0.66)	[14]
Prevalence of other oncogenic HPV in CIN2/3	0.34 (0.27; 0.41)	Uniform (0.27; 0.41)	[14]
Prevalence of HPV types 16 and 18 in CIN1	0.27 (0.21; 0.32)	Uniform (0.21; 0.32)	[14]
Prevalence of other oncogenic HPV in CIN1	0.28 (0.22; 0.33)	Uniform (0.22; 0.33)	[14]
Prevalence of HPV types 6 and 11 in CIN1	0.14 (0.11; 0.17)	Uniform (0.11; 0.17)	[14]
Prevalence of HPV types 6 and 11 in GW	0.95 (0.76; 1.00)	Uniform (0.76; 1.00)	[33]
Vaccine efficacy to HPV types 16 and 18 CC (Bivalent)	0.98 (0.94; 1.00)	Normal (0.94; 1.00)	[43, 44]
Vaccine efficacy to HPV types 16 and 18 CC (Quadrivalent)	0.98 (0.94; 1.00)	Normal (0.94; 1.00)	[45]
Vaccine efficacy to HPV types 16 and 18 CIN2/3 (Bivalent)	0.98 (0.97; 0.99)	Normal (0.97; 0.99)	[43, 44]
Vaccine efficacy to HPV types 16 and 18 CIN2/3 (Quadrivalent)	0.98 (0.97; 0.99)	Normal (0.97; 0.99)	[45]
Vaccine efficacy to HPV types16 and 18 CIN1 (Bivalent)	0.98 (0.97; 0.99)	Normal (0.97; 0.99)	[43, 44]
Vaccine efficacy to HPV types16 and 18 CIN1 (Quadrivalent)	0.98 (0.97; 0.99)	Normal (0.97; 0.99)	[45]
Vaccine efficacy to other oncogenic HPV CC (Bivalent)	0.68 (0.68; 0.68)	Normal (0.68; 0.68)	[47–50]
Vaccine efficacy to other oncogenic HPV CC (Quadrivalent)	0.33 (0.33; 0.33)	Normal (0.33; 0.33)	[46]
Vaccine efficacy to other oncogenic HPV CIN2/3 (Bivalent)	0.68 (0.68; 0.68)	Normal (0.68; 0.68)	[47–50]
Vaccine efficacy to other oncogenic HPV CIN2/3 (Quadrivalent)	0.33 (0.33; 0.33)	Normal (0.33; 0.33)	[46]
Vaccine efficacy to other oncogenic HPV CIN1 (Bivalent)	0.48 (0.48; 0.48)	Normal (0.48; 0.48)	[47–50]
Vaccine efficacy to other oncogenic HPV CIN1 (Quadrivalent)	0.23 (0.23; 0.23)	Normal (0.23; 0.23)	[46]
Vaccine efficacy to HPV types 6 and 11 (Quadrivalent)	0.98 (0.94; 1.00)	Normal (0.94; 1.00)	[66, 67]
GW incidence (10-79 years)	0.17% (0.089; 0.245)	Truncated Normal (Mean; range)	[31]
Costs			
Regular screening (Bs.F)	$2,943 (2,207; 3,678)	Uniform (2,207; 3,678)	see costs section
CIN1Onc newly detected (Bs.F)	$20,749 (15,562; 25,936)	Uniform (15,562; 25,936)	
CIN2/3 newly detected (Bs.F)	$22,230 (16,672; 27,787)	Uniform (16,672; 27,787)	
CIN1Onc det (Bs.F)	$15,284 (11,463; 19,105)	Uniform (11,463; 19,105)	
CIN2/3 det (Bs.F)	$28,355 (21,266; 35,444)	Uniform (21,266; 35,444)	
Cancer (Bs.F)	$273,788 (205,341; 342,234)	Uniform (205,341; 342,234)	
Genital Wart (Bs.F)	$9,568 (7,176; 11,960)	Uniform (7,176; 11,960)	
Vaccine bivalent (per dose) (USD)	$ 8.5	-	
Vaccine quadrivalent (per dose) USD; Scenarios 1-4	$ 8.5	-	
Vaccine quadrivalent (per dose) USD; Scenerios 5-8	$ 13.8	-	

*Abbreviations*: *NIS* National Institute of Statistics, *MoH* Ministry of Health, *VEF* Venezuelan bolívar fuerte, *US$* United States dollar, *Exp. Op* expert opinion, *BV* bivalent, *QV* quadrivalent, *CIN* cervical intraepithelial neoplasia, *CC* cervical cancer, *HPV* human papillomavirus, *VE* vaccine efficacy, *GW* genital wart, *y* year

#### Vaccinated population and coverage

Based on demographic data from the National Institute of Statistics of Venezuela, the model included a cohort of girls aged 11 years (n = 264,489) [4]. Vaccination coverage was assumed to reach 95% for a 3 dose scheme [5, 21], similar to what was previously assumed for an economic evaluation using the same model in Chile and the coverage observed for other vaccines in the country [28].

#### Epidemiological data

The general mortality rate by age in Venezuelan women was calculated from overall deaths and distribution of female population in 2011 [4, 6]. Number of deaths, overall mortality rate and mortality rate stratified by age were obtained from the National Mortality Yearbook of the Ministry of Health of Venezuela [6]. The age-specific incidence of high- and low-risk HPV was calculated based on data from a Venezuelan study [29] and adjusted to an age-specific tendency shown in a Chilean population-based study [30]. Data for GW incidence were scarce in the region; thus, the age-specific incidence of GW was matched to data reported in a large German population-based cohort study [31], in which the incidence is in average what is reported for many countries [32]. Data on type-specific distribution of HPV in CIN1, CIN2/3 and CC were obtained from Correnti et al. [14] and Sanchez-Lander et al. [15] Distribution of HPV 6 and 11 in GW was obtained from Avila et al. [33] Data on CIN1 and CIN2/3 natural history were obtained from Petry [34]. Information on treatment outcomes was provided by Melnikow et al. [35] and by local experts. Five-year CC cure rate for Venezuela was set on 41.5%, according to Rodriguez et al. [36] The coverage of regular screening of Pap was reported to be 35% every 3 years from age 25 to 64 years old [21, 37] and the percentage of positive Pap per screening campaign was 5% based on local experts opinion. Pap-smear-test-operative characteristics for CIN1 and CIN2/3 were obtained from Colantonio et al. [22]

#### Treatment costs

The model requires cost data for five health events’ treatments: screening, CIN1, CIN2/3, genital warts and cancer. In all cases we estimated expected costs using a micro-costing approach, which required to identify the resources used, the intensity of use and their unit costs. The list of resources were obtained from local literature [38] and their expected use was estimated using annual utilization rates obtained from administrative databases of *Dr. Luis Razetti Oncologic Institute* from Caracas, Venezuela, and validated through an expert opinion. All unit costs are of 2015 and were obtained from five local private health facilities, two of them of primary health care where 85% of the population with private coverage usually attend, while three with a high complexity level. Public tariffs were approximated from the last available *Gaceta Oficial* [39] and a private ambulatory health facility similar in costs. Finally, tariffs were weighted according to the distribution of utilization of public (74%) and private (26%) institutions [40]. When necessary costs were adjusted using the national Consumer Price Index [41] Details about unit costs, expected quantities and expected costs, treatment costs, vaccination costs per scenario and total costs (micro-costing) can be found in the Additional file 1.

#### Vaccine costs

Vaccine prices per person were calculated as the unit price of vaccines multiplied by the number of doses. Delivery costs were assumed to be equal for both vaccines and therefore were not considered in the analysis. Because there was uncertainty at the time the study was done about the price that Venezuela would pay once the vaccines were incorporated into the national program, two of the scenarios were projected considering equal price per dose at US$ 8.5 for each vaccine. The other scenario considered differential prices: US$ 8.5 per dose of the bivalent vaccine and US$ 13.79 per dose of the quadrivalent vaccine. These prices are based on last available Pan American Health Organization (PAHO) price agreements and projections for both vaccines, that is for 2013 [42].

#### Exchange rates

A key issue in the analysis is the election of the exchange rate. While expressing all values in USD or in VEF does not change results, the election of the rate does it because vaccines prices comes in USD and all the other costs are local estimations expressed in VEF. At the moment the analysis was done, there were in Venezuela several prevailing exchange rates and considering that it is not clear which one truly represents the opportunitiy costs of trading currencies, we decided to represent this uncertainty creating two scenarios for the most relevant rates: the exchange rate of 6.3 VEF per US$ (agreement No. 14, Ministry of Health of Venezuela vaccines purchase exchange rate) and the exchange rate of 170 VEF per US$ (agreement No. 33, regular citizens purchase exchange rate). Hence, choosing one rate over the other makes costs vary 27 times. Vaccine prices obtained from the PAHO Revolving Fund in US$.

#### Vaccine efficacy and cross-protection

For the estimation of vaccine effectiveness, HPV type-specific vaccine efficacy for both vaccines was considered as 98% against CC, CIN1 and CIN2/3 associated with HPV 16 and 18 with a lifelong duration [43–45]. The combined efficacy of the ten most frequent oncogenic HPV non-vaccine types (31,33,35,39,45,51,52,56,58 and 59) was considered jointly. Vaccine efficacy for these types, for the quadrivalent vaccine was 23.4% (95% Confident Interval – CI : 7.8-36.4) and 32.5% (95% CI: 6.0-51.9) for CIN1 and CIN2+, respectively [46]. Vaccine efficacy for the bivalent vaccine for these ten non-vaccine oncogenic types was 47.7% (95% CI 28.9 to 61.9) and 68.4 (95% CI: 45.7 to 82.4) for CIN1 and CIN2+, respectively [47–50]. Considering the latest evidence regarding the efficacy of the 2-dose scheme, case scenarios comparing the 2-dose schedule vs. the currently approved 3-dose schedule were developed; the same level and duration of efficacy and cross-protection were considered for both regimes [51–58]. In all scenarios, the duration of cross-protection for the base-case was set at 10 years, with a 5-year waning period.

#### Scenarios considered

For Venezuela, we needed to consider eight different plausible base-case scenarios, defined by the combination of a) two different official exchange rates, b) two number of doses per scheme, and c) two differential prices of vaccines (Table 2).

**Table 2:** Summary of base-case scenarios

Scenario	Vaccine Price in USD	Exchange rate	Dose scheme
1	BV: 8.5 - QV: 8.5	6.3	3 doses
2	BV: 8.5 - QV: 8.5	6.3	2 doses
3	BV: 8.5 - QV: 8.5	170	3 doses
4	BV: 8.5 - QV: 8.5	170	2 doses
5	BV: 8.5 - QV: 13.79	6.3	3 doses
6	BV: 8.5 - QV: 13.79	6.3	2 doses
7	BV: 8.5 - QV: 13.79	170	3 doses
8	BV: 8.5 - QV: 13.79	170	2 doses

For all these scenarios, the same cross protection and screening interval was assumed

*Abbreviations*: *BV* bivalent, *QV* quadrivalent, *VEF* Venezuelan bolívar fuerte, *US$* United States dollar

#### Outcomes

The present exercise explored health outcomes and disease-related costs for the different scenarios considered; we considered life years (LYs), QALYs, number of cervical cancer cases and deaths, number of new and recurrent genital warts, and total costs other than vaccination, including costs of screening, of CIN cases, warts and cancer cases. Another set of outcomes explored was the vaccine costs per scenario.

#### Discounting

The health benefits and costs were discounted at an annual rate of 5.0% based on guidelines recommendations for economic evaluations in LAC [59]. The effect of this discount rate on the outcomes was assessed in the different scenarios.

### Calibration

**Fig. 2:**
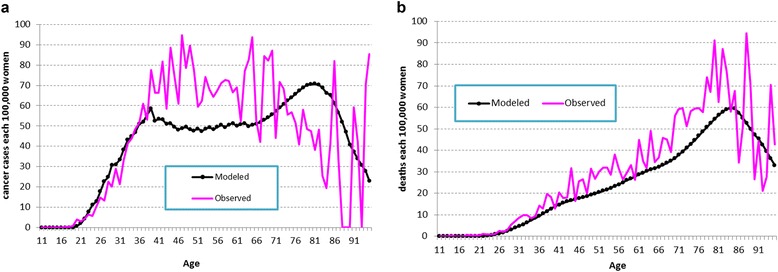
Model calibration. Observed vs. model predicted values. Incidence (**a**) and mortality (**b**) of invasive cervical cancer in Venezuela predicted by the model and observed by Globocan and National Health Statistics

The model was calibrated by modifying input parameters to match the model output to the data from vital statistics of Venezuela, keeping transition probabilities within pre-determined ranges of plausibility (Fig. 2). Age-specific CC incidence and mortality rates, as well as the overall number of cases and deaths estimated by the model were matched to observed vital statistics’data. On average, modeled event rates were within +/-10% of the observed event rates. Age-specific CC incidence and mortality were graphed and the resulting estimated and observed curves were visually explored to confirm a good fit. Since up-to-date cancer incidence data are likely to reflect a worse screening coverage prevailing in the past, the model was also calibrated using coverage rates representative of the Venezuelan situation 15 years ago, according to the consensus opinion of the group of authors.

### Deterministic and probabilistic sensitivity analysis

Parameter uncertainty was represented with one-way and multi-way sensitivity analyses. The objective in the first case was to identify the main drivers of results. Each parameter was varied separately from its base-case value according to a specific range (Table 1) which for all parameters were defined following three strategies according to the availability of information: i) using the reported confidence intervals when possible ii) approximating them using the percentage of variations reported in other studies [22, 60] iii) approximating them based on expert opinions.. The analysis was performed for the eight scenarios and summarized in tornado diagrams for costs and QALYs separately. Since the construction of the eight scenarios affects only costs, we included one tornado diagram for QALYs. (see Appendix) The probabilistic exercise was performed for each scenario to quantify the effect of uncertainty surrounding the model input parameters and to calculate probabilities of being cost-effective. In every case 1,000 simulations were generated to produce a distribution of the model results (Table 1) and was performed comparing both bivalent and quadrivalent vaccines vs. screening alone and bivalent vs. quadrivalent vaccines schemes. Simulations were plotted on the cost-effectiveness plane and used to construct cost-effectiveness acceptability curves (CEAC). These curves depict the probability of being the cost-effective option of the three (to have a higher monetary benefit) [61] for each intervention according to different units of GDP per capita as threshold values. The values the curves show can be interpreted as the probabilities of being the preferred option at different cost-effectiveness thresholds [62]. Only scenarios 2 and 4 (same vaccine price with 2-dose schemes) were presented for sensitivity analyses, and the rest can be found in the additional online material.

## Results

### Calibration

The model was calibrated to properly reflect the epidemiology of Venezuela. The model predicted rates of CC incidence and deaths closely matched to the incidence data for invasive CC reported by Globocan 2012 [63] and the mortality data for invasive CC reported by the National Mortality Yearbook of the Ministry of Health of Venezuela [6], as shown in Fig. 2. The difference between the observed and the modeled cumulative CC cases and deaths was below 10%.

For the cohort of 264,489 girls, the most important health outcomes and disease-related costs, discounted and undiscounted, are depicted in Table 3-A. These results show the differential gains between bivalent vaccine vs. no vaccination, quadrivalent vaccine vs. no vaccination and both vaccines against each other. Also, these results do not differ between scenarios, as their differences are mainly due to vaccine-related costs and exchange rates used for the different scenarios. Independently of the type of vaccine considered, there are gains in QALYs, life-years (LYs), CC cases and prevented deaths as compared to no vaccination, both considering discounted and undiscounted results. When comparing both vaccines against each other, the bivalent vaccine gains 3,544 undiscounted additional QALYs vs. the quadrivalent vaccine. Also, there are fewer CC cases and related deaths with the bivalent vaccine than with the quadrivalent for both undiscounted and discounted results. Regarding GWs, the quadrivalent prevented 18,469 new and recurrent GW cases (not discounted). Excluding the costs of vaccination, total medical costs for bivalent vaccine are slightly higher in the discounted scenario and lower in the undiscounted, when compared to the quadrivalent vaccine. The overall cost of the different vaccination programs for each scenario are presented in Table 3-B for the 2-dose scheme scenarios (2, 4) and in Additional file 1: Table S2 for the 3-dose scheme scenarios (1, 3, 5 and 7) and 2-dose scheme scenario with differential pricing for vaccines.

**Table 3:** Base-case analysis

	Results			Differences (Gains)		
	No vaccination (NV)	Quadrivalent (QV)	Bivalent (BV)	QV - NV	BV - NV	BV-QV
A: Health outcomes and disease-related costs						
Undiscounted						
LYs	18,351,285	18,395,244	18,398,735	43,959	47,451	3,491
QALYs	18,337,584	18,390,430	18,393,973	52,845	56,389	3,544
Cervical cancer cases	7,398	2,967	2,616	−4,431	−4,781	−350
Cervical cancer deaths	3,701	1,558	1,391	−2,143	−2,310	−168
Genital Warts (new and recurrent)	21,025	2,566	21,025	−18,459	0	18,459
Total cost other than vaccination^a^	11,295.9 VEF	7,109.9 VEF	7,107.3 VEF	−4,186.0 VEF	−4,188.6 VEF	−2.6 VEF
Costs of screening	4,323.0 VEF	4,396.1 VEF	4,383.3 VEF	73.1 VEF	60.3 VEF	−12.8 VEF
Costs of CIN 1	601.4 VEF	303.3 VEF	344.9 VEF	−298.1 VEF	−256.5 VEF	41.5 VEF
Costs of CIN 2/3	262.3 VEF	122.7 VEF	102.6 VEF	−139.6 VEF	−159.7 VEF	−20.1 VEF
Costs of warts	309.2 VEF	37.7 VEF	309.3 VEF	−271.4 VEF	0.1 VEF	271.6 VEF
Costs of cancer	5,800.1 VEF	2,250.1 VEF	1,967.3 VEF	−3,550.0 VEF	−3,832.7 VEF	−282.7 VEF
Discounted (5%)						
LYs	5,277,952	5,281,034	5,281,282	3,082	3,330	248
QALYs	5,276,066	5,280,461	5,280,552	4,395	4,486	91
Total cost other than vaccination^a^	2,435.2 VEF	1,602.7 VEF	1,672.5 VEF	−832.6 VEF	−762.8 VEF	69.8 VEF
Costs of screening	1,130.3 VEF	1,151.9 VEF	1,148.5 VEF	21.6 VEF	18.2 VEF	−3.5 VEF
Costs of CIN 1	167.1 VEF	82.8 VEF	91.8 VEF	−84.3 VEF	−75.4 VEF	9.0 VEF
Costs of CIN 2/3	79.5 VEF	35.2 VEF	28.6 VEF	−44.3 VEF	−50.9 VEF	−6.6 VEF
Costs of warts	136.7 VEF	16.7 VEF	136.7 VEF	−120.0 VEF	0.0 VEF	120.0 VEF
Costs of cancer	921.6 VEF	316.0 VEF	266.9 VEF	−605.6 VEF	−654.7 VEF	−49.1 VEF
B: Vaccine costs per scenario^a^						
2: Both vaccines = 8.5 US$; 2-dose scheme - FX 6.3	0 VEF	26.9 VEF	26.9 VEF	26.9 VEF	26.9 VEF	0 VEF
4: Both vaccines = 8.5 US$; 2-dose scheme - FX: 170	0 VEF	726.2 VEF	726.2 VEF	726.2 VEF	726.2 VEF	0 VEF
6: BV: 8.5 US$ QV: 13.79 US$; 2-dose scheme - FX 6.3	0 VEF	43.7 VEF	26.9 VEF	43.7 VEF	26.9 VEF	−16.7 VEF
8: BV: 8.5 US$ QV: 13.79 US$; 2-dose scheme - FX: 170	0 VEF	1,178.1 VEF	726.2 VEF	1,178.1 VEF	726.2 VEF	−451.9 VEF

Notes: A. Health outcomes and disease related costs (with and without discounting); B. Cost of vaccination (Scenarios 2, 4, 6 and 8)

*Abbreviations*: *NV* no vaccination, *QV* quadrivalent, *BV* bivalent, *FX* foreign exchange rate (VEF per US$), *CC* cervical cancer. (See scenarios 1, 3, 5, 7 in Additional file 1: Table S2), *VEF* Venezuelan bolívar fuerte, *QALYs* quality-adjusted life years, *LYs* life-years, *CIN* cervical intraepithelial neoplasia, *US$* United States dollar

^a^Costs are expressed in millions of VEF, 2015

Discounted results for incremental QALYs, costs and cost-effectiveness ratios between bivalent and quadrivalent vs. no vaccination strategies and vaccines against each other (considering different scenarios for the vaccine costs) are summarized in Table 4 and Additional file 1: Table S3. Whatever vaccine-cost scenario is considered, any vaccination strategy is cost-saving or cost-effective compared with the status quo (no vaccination) using a 1 GDP per capita threshold. When comparing the bivalent vs. the quadrivalent vaccines, ICERs showed that there were small differences between them for the first six scenarios; however, when different prices per dose are considered, under and at an exchange rate equal to 170 VEF per US$, the bivalent strategy is dominant (Scenarios 7 and 8).

**Table 4:** Total QALYs, total costs, and incremental cost-effectiveness ratios for scenarios 2, 4, 6 and 8^a^

Scenario	Quadrivalent *vs* No vaccination			Bivalent *vs* No vaccination			Bivalent *vs* Quadrivalent		
	∆QALYs	∆COSTS	ICER	∆QALYs	∆COSTS	ICER	∆QALYs	∆COSTS	ICER
Scenario									
2: Both vaccines = 8.5 US$; 2-dose scheme - FX 6.3	4,395	−805.7 US$	cost-saving	4,486	−735.9 VEF	cost- saving	91	69.8 VEF	0.77
4: Both vaccines = 8.5 US$; 2-dose scheme - FX: 170		−106.4 US$	cost- saving		−36.6 VEF	cost- saving		69.8 VEF	0.77
6: BV: 8.5 US$ QV: 13.79 US$; 2-dose scheme - FX 6.3		−788.9 US$	cost- saving		−735.9 VEF	cost- saving		53.0 VEF	0.58
8: BV: 8.5 US$ QV: 13.79 US$; 2-dose scheme - FX: 170		345.5 US$	0.08		−36.6 VEF	cost-saving		−382.1 VEF	cost-saving

Notes: Results for a cohort of 264,489 women (discount = 5%)

*Abbreviations*: *FX* exchange rate (VEF per US$), *QV* quadrivalent, *BV* bivalent. GDP per capita: 104,404 VEF. [68] (See scenarios 1, 3, 5, 7 in Additional file 1: Table S2), *QALYs* quality-adjusted life years, *ICER* incremental cost-effectiveness ratio, *VEF* Venezuelan bolívar fuerte, *US$* United States dollar

^a^Costs and ICERs are expressed in millions of VEF, 2015

### Deterministic sensitivity analyses

**Fig. 3:**
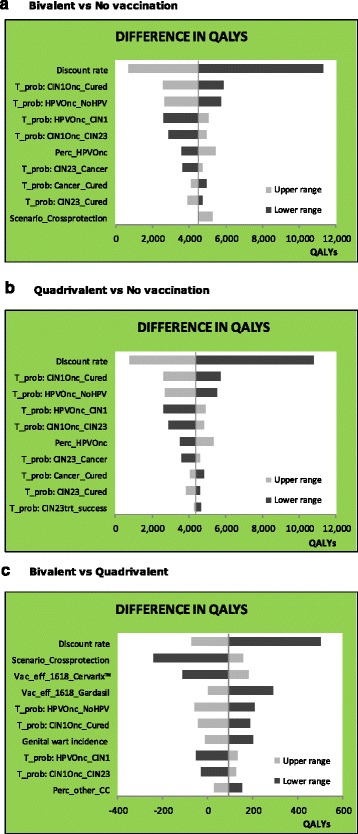
Deterministic Sensitivity Analysis: Tornado graph for QALYs differences. Discount rate = 5%. Abbreviations: T_pob: HPVlr_NoHPV = transition probability to regress from low risk HPV to no HPV; Scenario_Crossprotection = refers to the presence of lifetime cross-protection or no cross-protection at all; T_prob: HPVOnc_NoHPV = Transition probability to regress from infection with oncogenic HPV to no HPV; T_prob: HPVOnc_CIN1 = Transition probability to progress from infection with oncogenic HPV to CIN1; T_prob: CIN1Onc_CIN2/3 = Transition probability to progress from infection with oncogenic HPV and CIN1 to CIN2/3; T_prob: CIN2/3_Cancer = Transition probability to progress from CIN2/3 to cancer; T_prob: CIN1Onc_cured = Transition probability to cure from oncogenic HPV infection with CIN1; T_prob: CIN2/3_Cured = Transition probability to cure from CIN2/3; T_prob: Cancer_Cured = Transition probability to cure from cervical cancer; Vac_efficacy_1618_Cervarix = Vaccine efficacy for oncogenic types with quadrivalent vaccine; Vac_efficacy_1618_Gardasil = Vaccine efficacy for oncogenic types with bivalent vaccine; Vac_eff_other_Gardasil = Vaccine efficacy for non-vaccine oncogenic HPV types with bivalent vaccine; Perc_HPV_6_11 = Proportion of HPV 6 and 11 in genital warts; Perc_other_CC = Proportion of non-vaccine oncogenic HPV types among Cervical Cancer; Perc_HPVOnc = Proportion of HPV 16 and 18 among Cervical Cancer; QALYs: quality-adjusted life years; HPV: Human Papillomavirus

Tornado graphs for QALYs differences (Fig. 3) resulted in similar shapes for bivalent and quadrivalent vaccination strategies when compared to no vaccination. Discount rate was the main determinant due to differential horizon of costs and benefits, followed by transition probabilities. When comparing bivalent vs. quadrivalent strategies, the differences in QALYs (x-axis) were lower. Once again, the discount rate was the main determinant of variation. Cross-protection was the second parameter in importance and had a symmetrical and inverse impact over incremental QALY; that is, when no cross-protection was assumed, difference in QALY became lower and more negative, but when we considered a lifetime cross-protection, the impact was not so relevant in magnitude. These variations in assumptions do not change the conclusion that vaccines are cost-effective vs. screening alone.

**Fig. 4:**
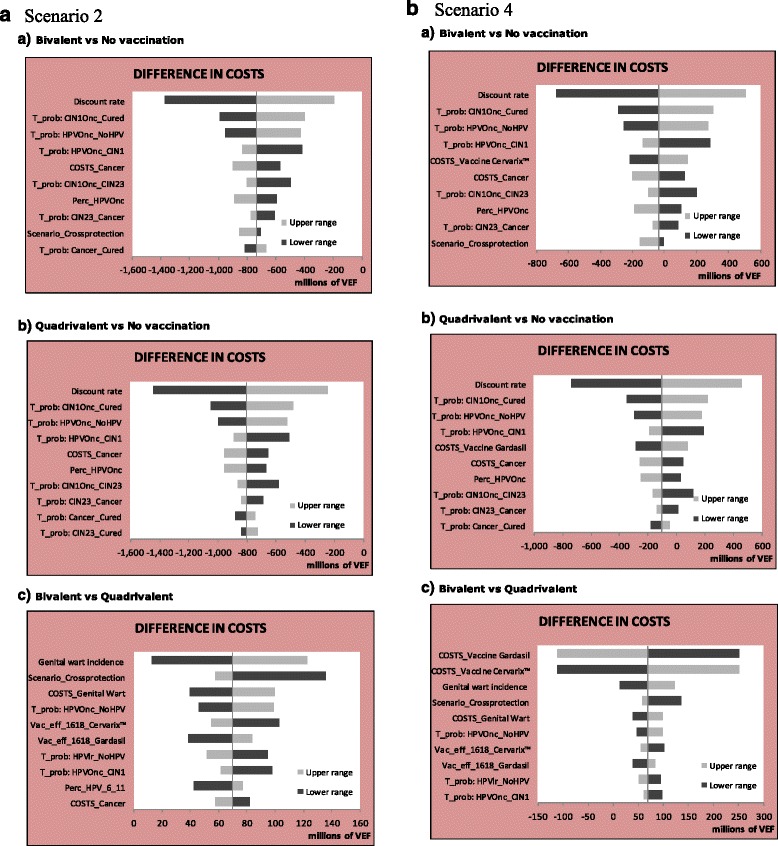
Deterministic sensitivity analysis – **a**) Scenario 2 Tornado graph for costs differences in scenario 2 (same vaccine price per dose of 8.5 US$, exchange rate of 6.3 VEF, scheme of 2 doses) **b**) Scenario 4 Tornado graph for costs differences in scenario 4 (same vaccine price per dose of 8.5 US$, exchange rate of 170 VEF, scheme of 2 doses). Discount rate = 5%. Abbreviations: COSTS_Genital Wart = Costs of genital warts management; COSTS_Cancer = Costs of cervical cancer management; COSTS_Vaccine cervarix = Cost of quadrivalent vaccine; COST_Vaccine Gardasil = Cost of bivalent vaccine; Scenario_Crossprotection = refers to the presence of lifetime cross-protection or no cross-protection at all; T_pob: HPVlr_NoHPV = transition probability to regress from low-risk HPV to no HPV; T_prob: HPVOnc_NoHPV = Transition probability to regress from infection with oncogenic HPV to no HPV; T_prob: HPVOnc_CIN1 = Transition probability to progress from infection with oncogenic HPV to CIN1; T_prob: CIN1Onc_CIN2/3 = Transition probability to progress from infection with oncogenic HPV and CIN1 to CIN2/3; T_prob: CIN2/3_Cancer = Transition probability to progress from CIN2/3 to cancer; T_prob: CIN1Onc_cured = Transition probability to cure from oncogenic HPV infection with CIN1; T_prob: CIN2/3_Cured = Transition probability to cure from CIN2/3; T_prob: Cancer_Cured = Transition probability to cure from cervical cancer; Vac_efficacy_1618_Cervarix = Vaccine efficacy for oncogenic types with quadrivalent vaccine; Vac_efficacy_1618_Gardasil = Vaccine efficacy for oncogenic types with bivalent vaccine; Vac_eff_other_Gardasil = Vaccine efficacy for non-vaccine oncogenic HPV types with bivalent vaccine; Perc_HPV_6_11 = Proportion of HPV 6 and 11 in genital warts; Perc_other_CC = Proportion of non-vaccine oncogenic HPV types among Cervical Cancer; Perc_HPVOnc = Proportion of HPV 16 and 18 among Cervical Cancer; HPV: Human Papillomavirus; VEF: Venezuelan bolívar fuerte; US$: United States dollar

Tornados graphs corresponding to the 2-dose scheme analysis (scenarios 2 and 4) are presented in Fig. 4 and 3-dose scheme analysis (scenarios 1, 3, 5 and 7) and scenarios with different vaccine prices with 2-dose scheme can be found in the Additional file 1. No parameter can modify the cost-saving results when any vaccine is compared with no vaccination in scenarios 2 and 6. There are several parameters than can modify the cost-saving result when any vaccine is compared with no vaccination in scenario 4 or when the bivalent vaccine is compared with no vaccination in scenario 8 (the most significant ones are the discount rate and the transition probabilities). Finally, only the discount rate can modify the result that the quadrivalent vaccine requires a positive investment in order to generate a cost-saving result (as shown for the bivalent vaccine) in scenario 8. The sensitivity analysis between vaccines showed that no parameter could modify the result that the quadrivalent vaccine was more costly than the bivalent vaccine in scenarios 2 and 6. The costs of both vaccines are the only parameters that can modify the result that the quadrivalent vaccine is more costly than the bivalent vaccine in scenario 4. Finally, no parameter can modify the result that the bivalent vaccine is less costly than the quadrivalent vaccine in scenario 8.

### Probabilistic sensitivity analyses

**Fig. 5:**
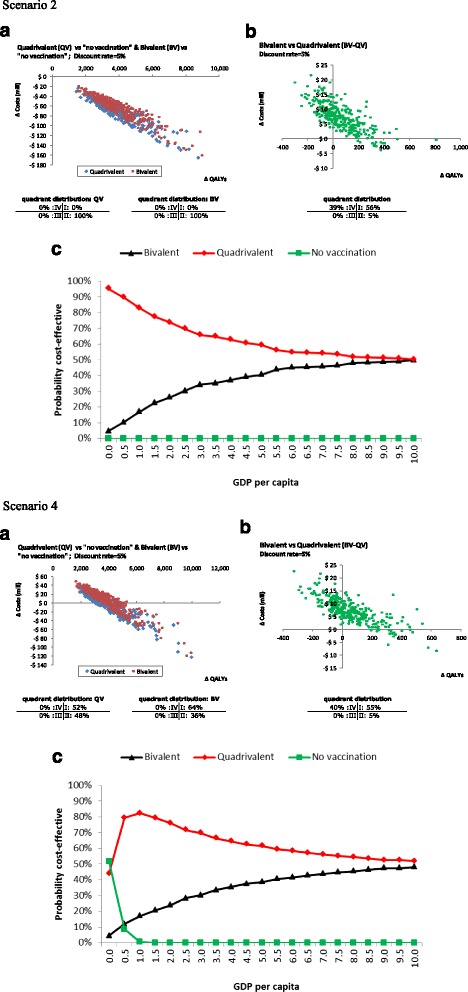
Probabilistic sensitivity analysis – Scatter plots for **a**) costs and **b**) QALYs differences, **c**) Cost-effectiveness acceptability curves (probability of being the most cost-effective intervention) for scenario 2 (same vaccine price per dose of 8.5 US$, exchange rate of 6.3 VEF, scheme of 2 doses) and scenario 4 (same vaccine price per dose of 8.5 US$, exchange rate of 170 VEF, scheme of 2 doses). Discount rate = 5%. Abbreviations: VEF: Venezuelan bolívar fuerte; US$: United States dollar; GDP: Gross Domestic Product

Cost-effectiveness plane and CEACs for interventions are presented in Fig. 5 for the scenarios 2 and 4. The other scenarios can be found in the Additional file 1. In each scenario, a first scatter-plot graph shows the results for both vaccines vs. no vaccination, and a second one shows the comparison of the vaccines against each other, with a 5% discount rate. PSAs confirm that both vaccines are cost-saving or cost-effective vs. no vaccination considering one GDP per capita (104,404 VEF), regardless of the discount applied (data not shown). Both the scatter-plots and the acceptability curves reflect the uncertainty from the three main variables that shape the different scenarios (i.e., exchange rates, number of doses and vaccine costs) and no superiority was detected of one vaccine over the other in scenarios 2, 4 and 6. In the scenarios considering a differential vaccine cost and an exchange rate of 170 VEF per US$ (scenario 8), the bivalent vaccine has 64% probabilities of being dominant and 100% probabilities of being cost-saving compared to the quadrivalent vaccine. The results are very sensitive to the discount rate (data not shown).

Acceptability curves for the studied scenarios indicate the probability that the intervention is cost-effective when compared to the alternatives, according to different thresholds or monetary values of QALYs expressed as units of GDP per capita. In all cases, the curves support the results suggested by the scatter plots. When comparing both vaccination interventions to screening only, all scenarios show that vaccines options are a cost-effective choice; however, in the comparison between vaccines, the analysis becomes diffuse. In one hand, the scenarios 2, 4 and 6 show the quadrivalent vaccine as the option with the higher probabilities of being the most cost-effective choice, with a threshold of at least 1 GDP per capita. The last scenario (scenario 8) on the other hand, shows the bivalent vaccine as having the highest probability of being the most cost-effective intervention. These results suggest, therefore, that the probability of being the most cost-effective intervention deeply depends on the scenario selected for analysis of exchange rate and vaccine prices.

## Discussion

Our study assessed the cost-effectiveness of HPV vaccination of 11-year-old girls in Venezuela vs. status quo (current screening strategy), from the healthcare system perspective, through adapting to this country a cohort model that has been used previously in other nations [18, 28]. Both vaccines currently available against HPV, the bivalent and the quadrivalent, were evaluated. The model was calibrated to reflect local HPV epidemiology in Venezuela.

Similarly to other studies, we faced difficulties to examine this issue in terms of the significant uncertainty of many model parameters, such as intervention, outcome costs, local epidemiological information and vaccine efficacy. Moreover, many countries are adopting a 2-dose scheme, even though the evidence is not as strong as for the 3-dose schedule.

Due to the current economic context in Venezuela, additional hurdles were to obtain adequate currency exchange rates to use (since the impact of the international price of the vaccine may vary greatly), as well as comparative vaccine prices. Though recent official prices showed that the bivalent vaccine was less costly, we considered an equal price per dose as well. So, instead of reporting a single base-case analysis, as it is usually the case in economic evaluations, and in order to incorporate these ranges of possible results, we needed to consider eight base-case scenarios including different exchange rates, different and equal vaccine prices and 2- and 3-dose schemes.

As a limitation of the model, vaccine delivery costs were not considered. However these costs do not alter results when comparing vacines, and in the scenarios versus no vaccination, the diferences observed in cost per QALY-gained are so important that the effect of including them would be within the range of what is considered cost-effective.

It is worthy to highlight that although the model was calibrated adequately with the epidemiologic value ranges reported by the Ministry of Health, the lack of accurate national information on some key parameters made us frequently use estimates from international literature and expert validation, similarly to previous studies. Our main findings were that HPV vaccination in 11-year-old girls in Venezuela would significantly reduce HPV-related diseases, and that they would be either cost-saving or cost-effective in all scenarios. Both the bivalent and quadrivalent vaccines showed similar results against no vaccination.

On six scenarios assessed, avoided costs were greater than costs of vaccination, resulting in cost-saving strategies. In two scenarios only – those using higher exchange rates and differential vaccines costs-vaccination was more costly than current screening. Nevertheless, in both cases, both vaccines were cost-effective, with costs per QALYs of less than 1 GDP per capita.

While the bivalent vaccine reduced a larger number of CC cases and deaths as compared with the quadrivalent vaccine, the quadrivalent reduced also the number of GWs. The net effect was a slight difference in LYs and QALYs in favor of the bivalent vaccine, though these results were highly dependent on the assumptions of the study, especially in cross-protection and incidence of GW.

It was clear and consistent that any vaccination strategy for HPV prevention would not only be beneficial for the health of women in Venezuela, but it would also be cost-saving or cost-effective, and thus a good intervention from the public health point of view. There was uncertainty regarding the relative cost-effectiveness of both vaccines. Accordingly, it is difficult to draw conclusions on the comparative performance of the different vaccines since the results are less clear and had significant heterogeneity in the tested scenarios: in some scenarios the bivalent vaccine was cost-saving or cost-effective compared to the quadrivalent, while in others it was clearly not.

## Conclusions

Despite uncertainty in Venezuela concerning costs data, exchange rates and epidemiological parameters, the main conclusion of the present study is that HPV vaccination provides significant health benefits, being either a cost-saving or a cost-effective strategy against the current practice of screening and no vaccination.

Given the uncertainties identified and because of the relatively similar overall benefit of both vaccines measured in LYs or QALYs, the decision of choosing one vaccine over another should take into consideration not only the cost-effectiveness, but also other aspects that differentiate both vaccines that can be valued in the local context.

## Additional file

Additional file 1: Presents the tornado graphs for deterministic sensitivity analyses and scatter plots for costs and QALYs/cost-effectiveness acceptability curves for probabilistic sensitivity analyses for scenarios 1, 3, 5 and 7 (3-dose schemes), and scenarios 2 and 4 (2-dose schemes). Also presents micro-costing details. (DOCX 698 kb)

## Abbreviations

CC: Cervical cancer
CEAC: Cost-effectiveness acceptability curves
CIN: Cervical intraepithelial neoplasia
DSA: Deterministic sensitivity analysis
GDP: Gross domestic product
GW: Genital warts
HPV: Human Papillomavirus
ICER: Incremental cost-effectiveness ratio
LAC: Latin America and the Caribbean region
LYs: Life-years
PAHO: Pan American Health Organization
Pap: Papanicolaou smear test
PSA: Probabilistic sensitivity analysis
QALYs: Quality-adjusted life years
VEF: Venezuelan bolívar fuerte
