# Supplementary material for: Health economic evaluation of Human Papillomavirus vaccines in women from Venezuela by a lifetime Markov cohort model
Source: BMC Public Health. 2017 Feb 2;17:152. doi: 10.1186/s12889-017-4064-7 (PMC5289055; doi:10.1186/s12889-017-4064-7)
Supplement: Additional file 1: — Presents the tornado graphs for deterministic sensitivity analyses and scatter plots for costs and QALYs/cost-effectiveness acceptability curves for probabilistic sensitivity analyses for scenarios 1, 3, 5 and 7 (3-dose schemes), and scenarios 2 and 4 (2-dose schemes). Also presents micro-costing details. (DOCX 698 kb) [file 12889_2017_4064_MOESM1_ESM.docx]

**Additional file 1**

**Supplementary Tables** 2

Supplementary Table 1. Weights used to create mean weighted tariffs for health care resources based on the distribution of medical care seek in Venezuela in six health facilities**.** 2

Supplementary Table 2. Total costs of vaccination and difference between interventions compared (No vaccination, Quadrivalent and Bivalent). Scenarios 1, 3, 5 and 7. Millions of bolívares fuertes (VEF) of 2015. Venezuela 3

Supplementary Table 3. Total QALYs, total costs, and incremental cost-effectiveness ratios for scenarios 1, 3, 5 and 7. Millions of bolívares fuertes (VEF) of 2015. Venezuela 4

Supplementary Table 4: Micro-costing after detection of cervical intraepithelial neoplasia grade 1 (CIN1), due to Oncogenic types. Bolívares fuertes (VEF), 2015. Venezuela 5

Supplementary Table 5: Micro-costing of newly detected cervical intraepithelial neoplasia grade 1(CIN1) due to Oncogenic types. Bolívares fuertes (VEF), 2015. Venezuela 6

Supplementary Table 6: Micro-costing of cervical intraepithelial neoplasia grade 2/3 (CIN2/3) detected. Bolívares fuertes (VEF), 2015. Venezuela 7

Supplementary Table 7: Micro-costing of cervical intraepithelial neoplasia grade 2/3 (CIN2/3) newly detected. Bolívares fuertes (VEF), 2015. Venezuela 8

Supplementary Table 8: Micro-costing of regular screening. Bolívares fuertes (VEF), 2015. Venezuela 9

Supplementary Table 9: Micro-costing of cervical cancer. Bolívares fuertes (VEF), 2015. Venezuela 10

Supplementary Table 10: Micro-costing of Genital Warts. Bolívares fuertes (VEF), 2015. Venezuela 12

Supplementary Table 11: List of unit costs used for analysis. Bolívares fuertes (VEF), 2015. Venezuela 13

**Deterministic sensitivity analyses – Tornado graphs** 15

**Probabilistic sensitivity analyses** 22

Supplementary Figure 5. Probabilistic sensitivity analysis – 22

## Table S1. Weights used to create mean weighted tariffs for health care resources based on the distribution of medical care seek in Venezuela in six health facilities**.**

| Health Facility 1  (less complex) | Health Facility 2 | Health Facility 3 | Health Facility 4 | Health Facility 5 | Health Facility 6  (most complex) |
| --- | --- | --- | --- | --- | --- |
| 74% | 26% | | | | |
|  | 85% | | 15% | | |
|  | 11% | 11% | 1% | 1% | 1% |

Source: adapted from National Health Service (2011)^[[1]](#footnote-2)^

## Table S2. Total costs of vaccination and difference between interventions compared (No vaccination, Quadrivalent and Bivalent). Scenarios 1, 3, 5 and 7. Millions of bolívares fuertes (VEF) of 2015. Venezuela

|  |  |  |  | **TOTAL COSTS**  **(millions of VEF)** | | |  | **COSTS DIFFERENCES**  **(millions of VEF)** | | |
| --- | --- | --- | --- | --- | --- | --- | --- | --- | --- | --- |
|  |  |  |  | No vaccination (NV) | Quadrivalent (QV) | Bivalent  (BV) |  | QV-NV | BV-NV | BV-QV |
| **VACCINE COSTS PER SCENARIO** | | |  |  |  |  |  |  |  |  |
|  | 1: Both vaccines = 8.5 US$; 3-dose scheme - FX: 6.3 | |  | 0 | 40.4 | 40.4 |  | 40.4 | 40.4 | 0 |
|  | 3: Both vaccines = 8.5 US$; 3-dose scheme - FX: 170 | |  | 0 | 1,089.2 | 1,089.2 |  | 1,089.2 | 1,089.2 | 0 |
|  | 5: BV: 8.5 US$ QV: 13.79 US$; 3-dose scheme - FX: 6.3 | |  | 0 | 65.5 | 40.4 |  | 65.5 | 40.4 | - 25.1 |
|  | 7: BV: 8.5 US$ QV: 13.79 US$; 3-dose scheme - FX: 170 | |  | 0 | 1,767.1 | 1,089.2 |  | 1,767.1 | 1,089.2 | - 677.9 |

Notes: Costs of vaccination (Scenarios 1, 3, 5 and 7). Costs are expressed in millions of VEF, 2015. NV: No vaccination, QV: Quadrivalent, BV: Bivalent, FX: Foreign exchange rate (VEF per US$), CC: Cancer cases; VEF; Venezuelan bolívar fuerte; US$: United States dollar

Table S3. Total QALYs, total costs, and incremental cost-effectiveness ratios for scenarios 1, 3, 5 and 7. Millions of bolívares fuertes (VEF) of 2015. Venezuela

| **Scenario** | | **Quadrivalent vs  No vaccination** | | |  | **Bivalent vs  No vaccination** | | |  | **Bivalent vs  Quadrivalent** | | |
| --- | --- | --- | --- | --- | --- | --- | --- | --- | --- | --- | --- | --- |
|  |  | ∆QALYs | ∆COSTS | ICUR |  | ∆QALYs | ∆COSTS | ICUR |  | ∆QALYs | ∆COSTS | ICUR |
| **Scenario** | |  |  |  |  |  |  |  |  |  |  |  |
|  | 1: Both vaccines = 8.5 US$; 3-dose scheme - FX: 6.3 | 4.395 | -792.2 | **cost saving** |  | 4.486 | -722.4 | **cost saving** |  | 91 | 69.8 | 0.77 |
|  | 3: Both vaccines = 8.5 US$; 3-dose scheme - FX: 170 |  | 256.6 | 0.06 |  |  | 326.4 | 0.07 |  |  | 69.8 | 0.77 |
|  | 5: BV: 8.5 US$ QV:13.79 US$; 3-dose scheme - FX: 6.3 |  | -767.1 | **cost saving** |  |  | -722.4 | **cost saving** |  |  | 44.7 | 0.49 |
|  | 7: BV: 8.5 US$ QV:13.79 US$; 3-dose scheme - FX: 170 |  | 934.5 | 0.21 |  |  | 326.4 | 0.07 |  |  | -608.1 | **cost saving** |
|  |  |  |  |  |  |  |  |  |  |  |  |  |

Notes: Results for a cohort of 264,489 women (discount rate = 5%). Costs and ICURs are expressed in millions of VEF, 2015. FX: exchange rate (VEF per US$); QALYs: quality-adjusted life years; VEF; Venezuelan bolívar fuerte; US$: United States dollar; ICURs: Incremental Cost Utility Ratio; GDP per capita 2015: 0.16 million of Bolivars.^[[2]](#footnote-3)^

## Table S4: Micro-costing after detection of cervical intraepithelial neoplasia grade 1 (CIN1), due to Oncogenic types. Bolívares fuertes (VEF), 2015. Venezuela

| Resources | Quantities | Resource use | Unit cost (4) | Total cost |
| --- | --- | --- | --- | --- |
| Medical consultation with gynecologist (1) (3) | 3 | 80% | 684.81 | 1,643.55 |
| Biopsy (1) (3) | 1 | 80% | 561.31 | 449.05 |
| Cytology (1) (3) | 2 | 80% | 366.59 | 586.54 |
| Colposcopy (1) | 2 | 80% | 659.47 | 1,055.15 |
| Cryotherapy (2) (3) | 1 | 30% | 3,499.90 | 1,049.97 |
| Electrosurgical excision with loop (LEEP) (1) (3) | 1 | 30% | 34,999.01 | 10,499.70 |
|  |  |  |  | **15,283.96** |
| Notes: (1) Source of quantities: Medina Francisco et al. Cáncer de cuello uterino. Consenso Nacional para el diagnóstico y tratamiento 2010. Rev Venez Oncol 2011, (2) Source of quantities: Cortiñas Paula, Centeno Indira, Sánchez Lander Jorge, Martin David. Reunión de Consenso en Virus de Papiloma Humano 2008. Gac Méd Caracas 1009; (3) Source of resource use data: Consulta Instituto de Oncología Dr. Luis Razetti; (4) See supplementary Table 11 | | | | |

## Table S5: Micro-costing of newly detected cervical intraepithelial neoplasia grade 1(CIN1) due to Oncogenic types. Bolívares fuertes (VEF), 2015. Venezuela

| Resources | Quantities | Resource use | Unit cost (4) | Total cost |
| --- | --- | --- | --- | --- |
| Medical consultation with gynecologist (1) (3) | 3 | 70% | 684.81 | 1,438.11 |
| Cytology (1) (3) | 1 | 70% | 366.59 | 256.61 |
| Colposcopy (1) | 1 | 70% | 659.47 | 461.63 |
| Biopsy (1) (3) | 1 | 70% | 561.31 | 392.92 |
| Cryotherapy (2) (3) | 1 | 20% | 3,499.90 | 699.98 |
| Electrosurgical excision with loop (LEEP) (1) (3) | 1 | 50% | 34,999.01 | 17,499.51 |
|  |  |  |  | **20,748.75** |
| Notes: (1) Source of quantities: Medina Francisco et al. Cáncer de cuello uterino. Consenso Nacional para el diagnóstico y tratamiento 2010. Rev Venez Oncol 2011, (2) Source of quantities: Cortiñas Paula, Centeno Indira, Sánchez Lander Jorge, Martin David. Reunión de Consenso en Virus de Papiloma Humano 2008. Gac Méd Caracas 1009; (3) Source of resource use data: Consulta Instituto de Oncología Dr. Luis Razetti; (4) See supplementary Table 11 | | | | |

## Table S6: Micro-costing of cervical intraepithelial neoplasia grade 2/3 (CIN2/3) detected. Bolívares fuertes (VEF), 2015. Venezuela

| Resources | Quantities (1) | Resource use (2) | Unit cost (3) | Total cost |
| --- | --- | --- | --- | --- |
| Medical consultation with gynecologist | 3 | 80% | 684.81 | 1,643.55 |
| Cytology | 1 | 80% | 366.59 | 293.27 |
| Colposcopy | 2 | 80% | 659.47 | 1,055.15 |
| Biopsy | 1 | 80% | 561.31 | 449.05 |
| Electrosurgical excision with loop (LEEP) | 1 | 60% | 34,999.01 | 20,999.41 |
| Conization with cold scalpel | 1 | 30% | 13,048.27 | 3,914.48 |
|  |  |  |  | **28,354.90** |
| Notes: (1) Source of quantities: Medina Francisco et al. Cáncer de cuello uterino. Consenso Nacional para el diagnóstico y tratamiento 2010. Rev Venez Oncol 2011, (2) Source of resource use data: Consulta Instituto de Oncología Dr. Luis Razetti; (3) See supplementary Table 11 | | | | |

## Table S7: Micro-costing of cervical intraepithelial neoplasia grade 2/3 (CIN2/3) newly detected. Bolívares fuertes (VEF), 2015. Venezuela

| Resources | Quantities (1) | Resource use (2) | Unit cost (3) | Total cost |
| --- | --- | --- | --- | --- |
| Medical consultation with gynecologist | 3 | 70% | 684.81 | 1,438.11 |
| Cytology | 1 | 70% | 366.59 | 256.61 |
| Colposcopy | 2 | 70% | 659.47 | 923.25 |
| Biopsy | 1 | 70% | 561.31 | 392.92 |
| Electrosurgical excision with loop (LEEP) | 1 | 40% | 34,999.01 | 13,999.60 |
| Conization with cold scalpel | 1 | 40% | 13,048.27 | 5,219.31 |
|  |  |  |  | **22,229.80** |
| Notes: (1) Source of quantities: Medina Francisco et al. Cáncer de cuello uterino. Consenso Nacional para el diagnóstico y tratamiento 2010. Rev Venez Oncol 2011, (2) Source of resource use data: Consulta Instituto de Oncología Dr. Luis Razetti; (3) See supplementary Table 11 | | | | |

## Table S8: Micro-costing of regular screening. Bolívares fuertes (VEF), 2015. Venezuela

| Resources | Quantities (1) | Resource use (2) | Unit cost (3) | Total cost |
| --- | --- | --- | --- | --- |
| Medical consultation with gynaecologist | 2 | 100% | 1,240.00 | 2,480.00 |
| Papanicolaou | 1 | 100% | 462.50 | 462.50 |
|  |  |  |  | **2,942.50** |
| Notes: (1) Source of quantities: Medina Francisco et al. Cáncer de cuello uterino. Consenso Nacional para el diagnóstico y tratamiento 2010. Rev Venez Oncol 2011, (2) Source of resource use data: Consulta Instituto de Oncología Dr. Luis Razetti; (3) See supplementary Table 11 | | | | |

## Table S9: Micro-costing of cervical cancer. Bolívares fuertes (VEF), 2015. Venezuela

| Resources | | Quantities | Resource use | Unit cost (4) | Total cost |
| --- | --- | --- | --- | --- | --- |
| **Cáncer Cervical - stage IA1** | |  | 3.5% |  |  |
|  | Day of hospitalization (1) (3) | 2 | 70% | 3,704.59 | 5,186.43 |
|  | Hysterectomy, simple (1) (3) | 1 | 40% | 92,720.87 | 37,088.35 |
|  | Conization with ASA or cold (1) (3) | 1 | 30% | 13,048.27 | 3,914.48 |
|  | Chest x-ray (1) (3) | 1 | 70% | 1,073.83 | 751.68 |
|  | Consultation with oncologist / gynecologist (1) (3) | 3 | 70% | 684.81 | 1,438.11 |
|  | Laboratory (3) | 1 | 70% | 2,110.86 | 1,477.61 |
|  | Preoperative evaluation (consultation) (3) | 1 | 70% | 2,043.20 | 1,430.24 |
|  | Magnetic resonance imaging of abdomen and pelvis (3) | 1 | 50% | 8,007.35 | 4,003.67 |
|  | Biopsy (3) | 1 | 70% | 561.31 | 392.92 |
|  | Subtotal |  |  |  | 55,683.49 |
| **Cáncer Cervical - stage IA2, IB1, IIA1** | |  | 36.8% |  |  |
|  | Day of hospitalization (1) (3) | 2 | 70% | 3,704.59 | 5,186.43 |
|  | Radical hysterectomy (1) (3) | 1 | 70% | 92,720.87 | 64,904.61 |
|  | Biopsy (1) (3) | 1 | 70% | 561.31 | 392.92 |
|  | Chest x-ray (1) (3) | 1 | 70% | 1,073.83 | 751.68 |
|  | Consultation with oncologist / gynecologist (1) (3) | 3 | 70% | 684.81 | 1,438.11 |
|  | MRI of the abdomen and pelvis (1) (3) | 1 | 50% | 8,007.35 | 4,003.67 |
|  | Subtotal |  |  |  | 76,677.42 |
| **Cáncer Cervical - stage IB2, IIA2, III y IV** | |  | 59.6% |  |  |
|  | Chest x-ray (per study) (1) (3) | 1 | 80% | 1,073.83 | 859.07 |
|  | Cystoscopy (per study) (1) (3) | 1 | 80% | 2,382.27 | 1,905.81 |
|  | Rectosigmoidoscopy (per study) (1) (3) | 1 | 80% | 2,926.45 | 2,341.16 |
|  | Chemotherapy (per session) (1) (3) | 6 | 80% | 61,706.44 | 49,365.15 |
|  | Radiotherapy (per session) (1) (3) | 25-30 | 80% | 265,144.02 | 212,115.22 |
|  | Brachytherapy (per session) (3) | 4 | 80% | 37,565.61 | 120,209.94 |
|  | Consultation with a medical oncologist (3) | 8 | 80% | 1,060.58 | 6,787.69 |
|  | Consultation with readiotherapist (3) | 6 | 80% | 1,590.86 | 7,636.15 |
|  | Hospitalization due to complications (per day) (3) | 5 | 20% | 3,704.59 | 3,704.59 |
|  | Nuclear magnetic resonance (3) | 1 | 10% | 4,003.67 | 400.37 |
|  | Abdominoplasty (3) | 1 | 60% | 5,168.88 | 3,101.33 |
|  | Subtotal |  |  |  | 408,426.47 |
| Total (weighted average) | |  |  |  | **273,787.53** |
| Notes: (1) Source of quantities: Medina Francisco et al. Cáncer de cuello uterino. Consenso Nacional para el diagnóstico y tratamiento 2010. Rev Venez Oncol 2011, (2) Source of quantities: Cortiñas Paula, Centeno Indira, Sánchez Lander Jorge, Martin David. Reunión de Consenso en Virus de Papiloma Humano 2008. Gac Méd Caracas 1009; (3) Source of resource use data: Instituto de Oncología Dr. Luis Razetti; (4) See supplementary Table 11 | | | | | |

## Table S10: Micro-costing of Genital Warts. Bolívares fuertes (VEF), 2015. Venezuela

| Resources | Quantities (1) | Resource use (1) | Unit cost (2) | Total cost |
| --- | --- | --- | --- | --- |
| Medical consultation with specialist doctor | 3 | 100% | 616.57 | 1,849.70 |
| Pap smear | 1 | 90% | 366.59 | 329.93 |
| Colposcopy | 3 | 60% | 659.47 | 1,187.04 |
| Imiquimod self-treatment | 1 | 60% | 841.04 | 504.62 |
| Cryotherapy (session) | 1 | 20% | 3,499.90 | 699.98 |
| 15-15% Podofilin / cauterization or excision (per session) | 3 | 10% | 599.06 | 179.72 |
| Surgery | 1 | 25% | 19,266.40 | 4,816.60 |
|  |  |  |  | **9,567.58** |
| Notes: (1) Source of quantities and resource use: Instituto de Oncología Dr. Luis Razetti; (2) See supplementary Table 11 | | | | |

## Table S11: List of unit costs used for analysis. Bolívares fuertes (VEF), 2015. Venezuela

| **Item** | **Unit cost  (VEF)** |
| --- | --- |
| 15-25% Podofilin / cauterization or excision | 599 |
| Abdominoplasty | 5,169 |
| Biopsy | 529 |
| Brachytherapy | 37,566 |
| Chemotherapy drugs | 61,706 |
| Colposcopy | 659 |
| Consultation with Gynecologist | 685 |
| Consultation with Gynecologist II | 1,240 |
| Consultation with radiotherapist | 1,591 |
| Consultation with specialist doctor | 617 |
| Conventional surgery (cold scalpel) (neck conization) | 13,048 |
| Cryotherapy | 3,500 |
| Cystoscopy | 2,382 |
| External Radiotherapy | 265,144 |
| Hospitalization day | 3,705 |
| Imiquimod self-treatment: 5% cream (80%) or 3.75% cream (20%) | 841 |
| Magnetic resonance imaging of abdomen and pelvis | 8,007 |
| Magnetic resonance, with contrast | 4,004 |
| Oncologist Consultation | 1,061 |
| Papanicolaou / Cytology | 367 |
| Preoperative evaluation (consultation) | 2,043 |
| Preoperative profile | 2,111 |
| Radical Hysterectomy | 92,721 |
| Rectoscopy | 2,926 |
| Surgery Electrosurgery (Ib, A) | 9,752 |
| Surgery Radiofrequency (electrosurgery or Leep) | 34,999 |
| Torax X-ray, two projections | 1,074 |
| Notes: Unit costs were obtained from six local health facilities. Values are weighted averages according to the information of supplementary table 1 and are expressed in 2015 Bolívares Fuertes (VEF) | |

# **Deterministic sensitivity analyses – Tornado graphs**

**Abbreviations**: *COSTS_Genital Wart = Costs of genital warts management; COSTS_Cancer = Costs of cervical cancer management; COSTS_Vaccine Cervarix™ = Costs of quadrivalent vaccine; COSTS_Vaccine Gardasil = Costs of bivalent vaccine; T_prob: HPVlr_NoHPV = transition probability to regress from low-risk HPV to no HPV; T_prob: HPVOnc_NoHPV = Transition probability to regress from infection with oncogenic HPV to no HPV; T_prob: HPVOnc_CIN1 = Transition probability to progress from infection with oncogenic HPV to CIN1; T_prob: CIN1Onc_CIN23 = Transition probability to progress from infection with oncogenic HPV and CIN1 to CIN2/3; T_prob: CIN23_Cancer = Transition probability to progress from CIN2/3 to cancer; T_prob: CIN1Onc_cured = Transition probability to cure from oncogenic HPV infection with CIN1; T_prob: CIN23_Cured = Transition probability to cure from CIN2/3; T_prob: Cancer_Cured = Transition probability to cure from cervical cancer; Vac_eff_1618_Cervarix™ = Vaccine efficacy for oncogenic types with quadrivalent vaccine; Vac_eff_1618_Gardasil = Vaccine efficacy for oncogenic types with bivalent vaccine; Vac_eff_other_Gardasil = Vaccine efficacy for non-vaccine oncogenic HPV types with bivalent vaccine; Perc_HPV_6_11 = Proportion of HPV 6 and 11 in genital warts; Perc_other_CC = Proportion of non-vaccine oncogenic HPV types among Cervical Cancer; Perc_HPVOnc = Proportion of HPV 16 and 18 among Cervical Cancer.*

Figure S1 above. Deterministic sensitivity analysis – Scenario 1: Tornado graph for costs differences in scenario 1 (same vaccine price per dose of 8.5 US$, exchange rate of 6.3 US$, scheme of 3 doses). Discount rate = 5%.

Figure S2 above. Deterministic sensitivity analysis – Scenario 3: Tornado graph for costs differences in scenario 3 (same vaccine price per dose of 8.5 USD, exchange rate of 170 US$, scheme of 3 doses). Discount rate = 5%.

Figure S3 above. Deterministic sensitivity analysis – Scenario 5: Tornado graph for costs differences in scenario 5 (Vaccine price per dose of 8.5 US$ for bivalent and 12 US$ for quadrivalent, exchange rate of 6.3 US$, scheme of 3 doses). Discount rate = 5%.

Figure S4 above. Deterministic sensitivity analysis – Scenario 7: Tornado graph for costs differences in scenario 7 (Vaccine price per dose of 8.5 US$ for bivalent and 12 US$ for quadrivalent, exchange rate of 170 US$, scheme of 3 doses). Discount rate = 5%.

Figure S5 below. Deterministic sensitivity analysis – Scenario 6: Tornado graph for costs differences in scenario 7 (Vaccine price per dose of 8.5 US$ for bivalent and 13.79 US$ for quadrivalent, exchange rate of 170 US$, scheme of 2 doses). Discount rate = 5%.

A) Scenario 6

Figure S6 below. Deterministic sensitivity analysis – Scenario 8: Tornado graph for costs differences in scenario 7 (Vaccine price per dose of 8.5 US$ for bivalent and 13.79 US$ for quadrivalent, exchange rate of 170 US$, scheme of 2 doses). Discount rate = 5%.

B) Scenario 8

# **Probabilistic sensitivity analyses**

## Figure S5. Probabilistic sensitivity analysis –

Scatter plots for **A.)** costs and **B.)** QALYs differences, **C.)** Cost-effectiveness acceptability curves (probability of being the most cost-effective intervention) for scenario 1 (same vaccine price per dose of 8.5 US$, exchange rate of 6.3 US$, scheme of 3 doses), scenario 3 (same vaccine price per dose of 8.5 US$, exchange rate of 170 US$, scheme of 3 doses), scenario 5 (vaccine price per dose of 8.5 US$ for bivalent and 13.79 US$ for quadrivalent, exchange rate of 6.3 US$, scheme of 3 doses) and scenario 7 (vaccine price per dose of 8.5 US$ for bivalent and 13.79 US$ for quadrivalent, exchange rate of 170 US$, scheme of 3 doses). Discount rate = 5%. Also shown scenarios with 2 doses: Scenario 6 (vaccine price per dose of 8.5 US$ for Bivalent and 13.79 US$ for Quadrivalent, exchange rate of 6.3 VEF, scheme of 2 doses) and scenario 8 (vaccine price per dose of 8.5 US$ for bivalent and 13.79 US$ for quadrivalent, exchange rate of 170 VEF, scheme of 2 doses). Discount = rate 5%.

Scenario 1

| A) | B) |
| --- | --- |

C)


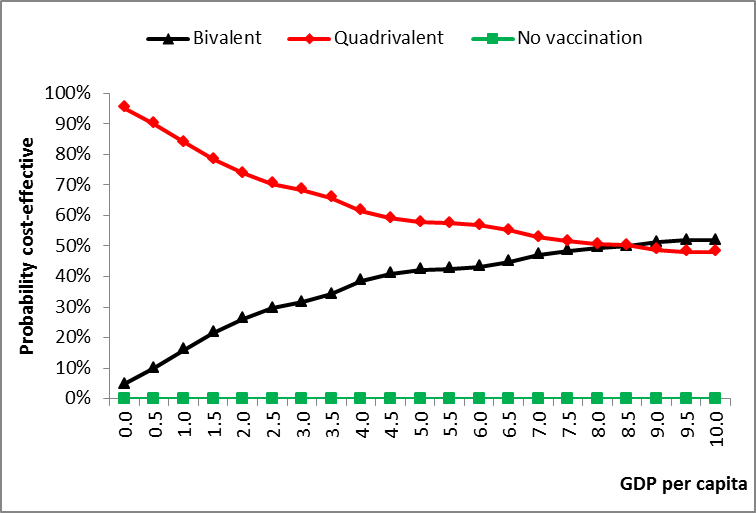


Scenario 3

| A) | B) |
| --- | --- |

C)


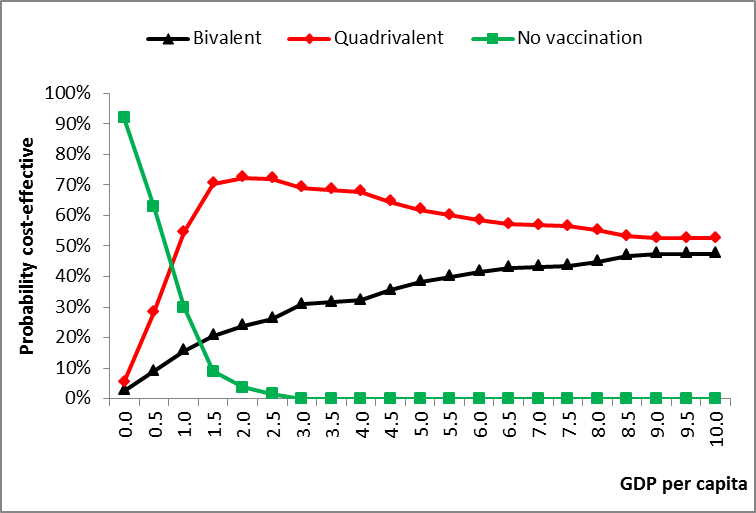


Scenario 5

| A) | B) |
| --- | --- |

C)


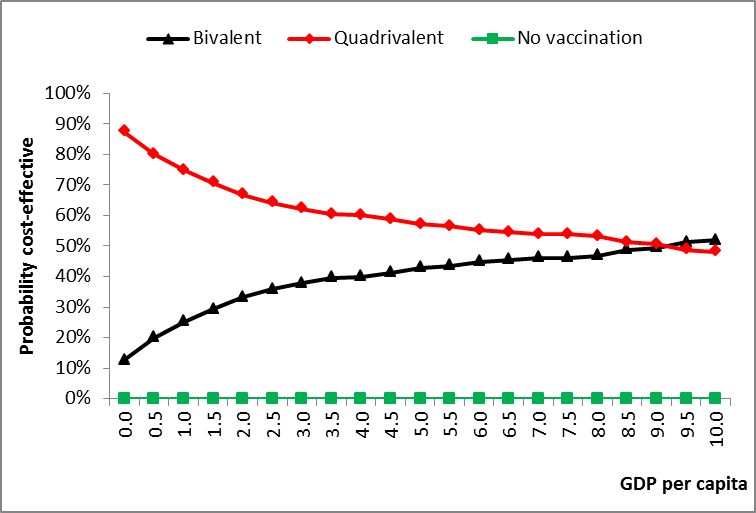


Scenario 7

| A) | B) |
| --- | --- |

C)


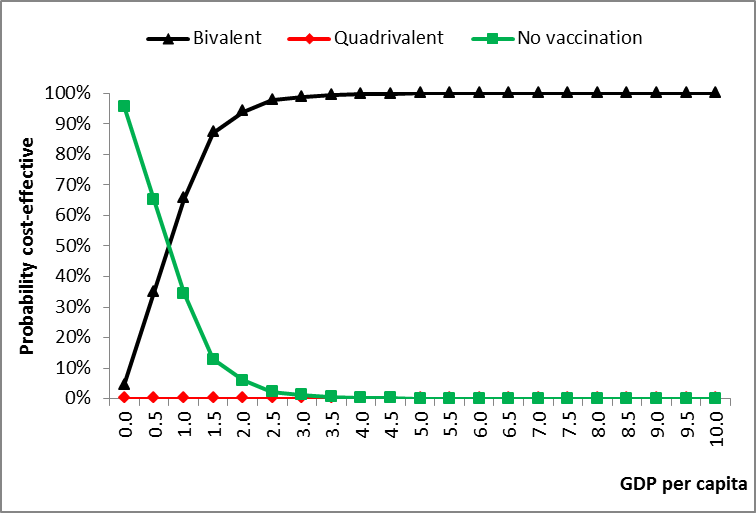


Scenario 6

| A) | B) |
| --- | --- |

C)


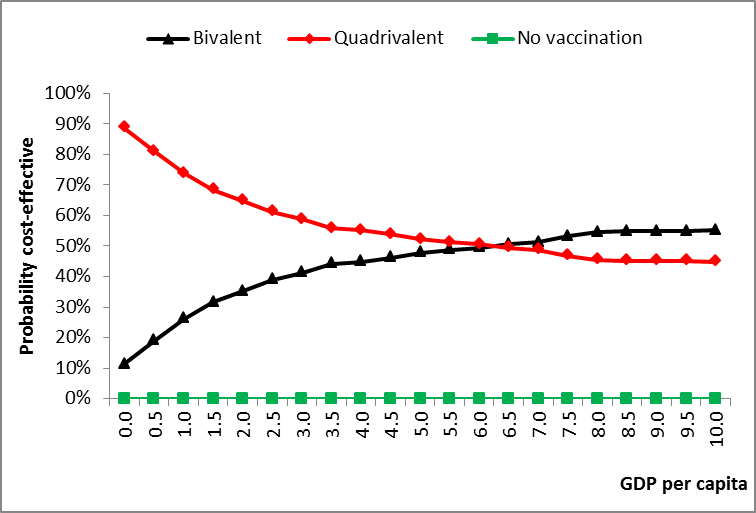


Scenario 8

| A) | B) |
| --- | --- |

C)


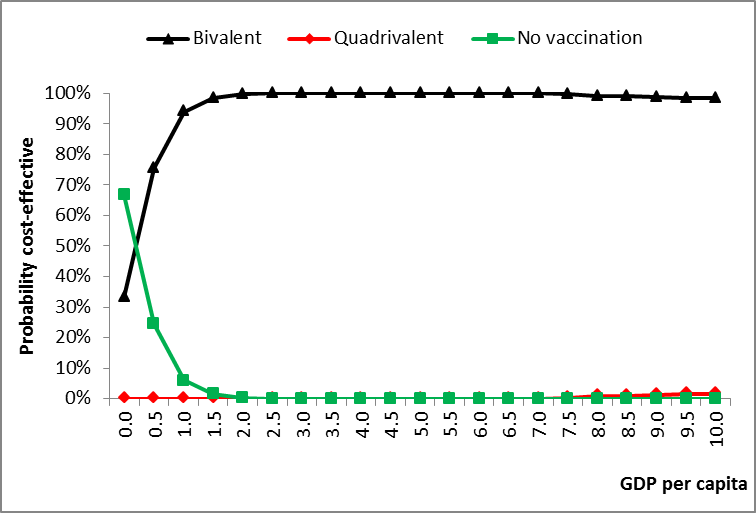


1. National Health Services, UK. Technical Guide to the formulae for 2014-15 and 2015- 16 revenue allocations to Clinical Commissioning Groups and Area Teams. <https://www.england.nhs.uk/wp-content/uploads/2014/03/tech-guide-rev-allocs.pdf> Accessed Nov 2, 2015 [↑](#footnote-ref-2)
2. GDP Per Capita (Current Prices, National Currency) Data for All Countries. 2015. EconomyWatch.com's Econ Stats database. http://www.economywatch.com/economic-statistics/economic-indicators/GDP_Per_Capita_Current_Prices_National_Currency/ Accessed November 2, 2015 [↑](#footnote-ref-3)
